# The genome sequence of a leaf beetle, *Chrysolina haemoptera *(Linnaeus, 1758)

**DOI:** 10.12688/wellcomeopenres.22887.1

**Published:** 2024-08-07

**Authors:** Brian Levey, Natural History Museum Genome Acquisition Lab, Darwin Tree of Life Barcoding collective, Wellcome Sanger Institute Tree of Life Management, Samples and Laboratory team, Wellcome Sanger Institute Scientific Operations: Sequencing Operations, Wellcome Sanger Institute Tree of Life Core Informatics team, Tree of Life Core Informatics collective, Darwin Tree of Life Consortium

**Affiliations:** 1 Amgueddfa Cymru, Cardiff, Wales, UK

**Keywords:** Chrysolina haemoptera, leaf beetle, genome sequence, chromosomal, Coleoptera

## Abstract

We present a genome assembly from a female leaf beetle, *Chrysolina haemoptera* (Arthropoda; Insecta; Coleoptera; Chrysomelidae). The total length of the genome sequence is 718.30 megabases. Most of the assembly is scaffolded into 20 chromosomal pseudomolecules, including the X sex chromosome. The mitochondrial genome has also been assembled and is 17.87 kilobases long. Gene annotation of this assembly on Ensembl identified 12,298 protein-coding genes.

## Species taxonomy

Eukaryota; Opisthokonta; Metazoa; Eumetazoa; Bilateria; Protostomia; Ecdysozoa; Panarthropoda; Arthropoda; Mandibulata; Pancrustacea; Hexapoda; Insecta; Dicondylia; Pterygota; Neoptera; Endopterygota; Coleoptera; Polyphaga; Cucujiformia; Chrysomeloidea; Chrysomelidae; Chrysomelinae; Chrysomelini; *Chrysolina*; *Chrysolina haemoptera* (Linnaeus, 1758) (NCBI:txid75519).

## Background

Chrysomelidae (leaf beetles) are one of the most diverse clades within Coleoptera, currently containing over 40,000 species, of which about 250 are recorded from Britain and Ireland ( Duff, 2018). The genus *Chrysolina* (Motschulsky, 1860) is one of the most speciose genera within the subfamily Chrysomelinae, with almost 500 species currently considered valid ( Bieńkowski, 2007; Kippenberg, 2010). Nineteen *Chrysolina* species occur in Britain and Ireland ( Duff, 2018).

*C. haemoptera* is a 5–9 mm long, dull, blue-black specimen with no metallic reflection. It is very similar in appearance to *C. oricalcia*, but can be distinguished by its pronotum, which has no lateral longitudinal furrows, its straight sides, and the cone-like narrowing towards the front ( UK Beetle Recording, 2024).

*C. haemoptera* is widely distributed across Europe and parts of Asia. It is found in various countries including France, Germany, Poland, Italy, and extending into Western Asia ( GBIF Secretariat, 2024). In the United Kingdom, *C. haemoptera* is classified is listed as Scarce (Notable B) ( UK Beetle Recording, 2024). It can be found in southern and eastern England, usually, but not always coastal. The main host plant is plantain ( *Plantago* spp.).

Three other full genome sequences for the genus *Chrysolina* have been generated by the Darwin Tree of Life project to date: *C. americana* (GCA_958502065.1) and *C. oricalcia* (GCA_944452925.2) ( Sivell *et al.*, 2023) and *C. graminis (*GCA_964197785.1). Here we present the first chromosomally complete genome sequence for *C. haemoptera*, based on a female specimen from Loe Bar, Porthleven, England, UK.

## Genome sequence report

**Figure 1.**
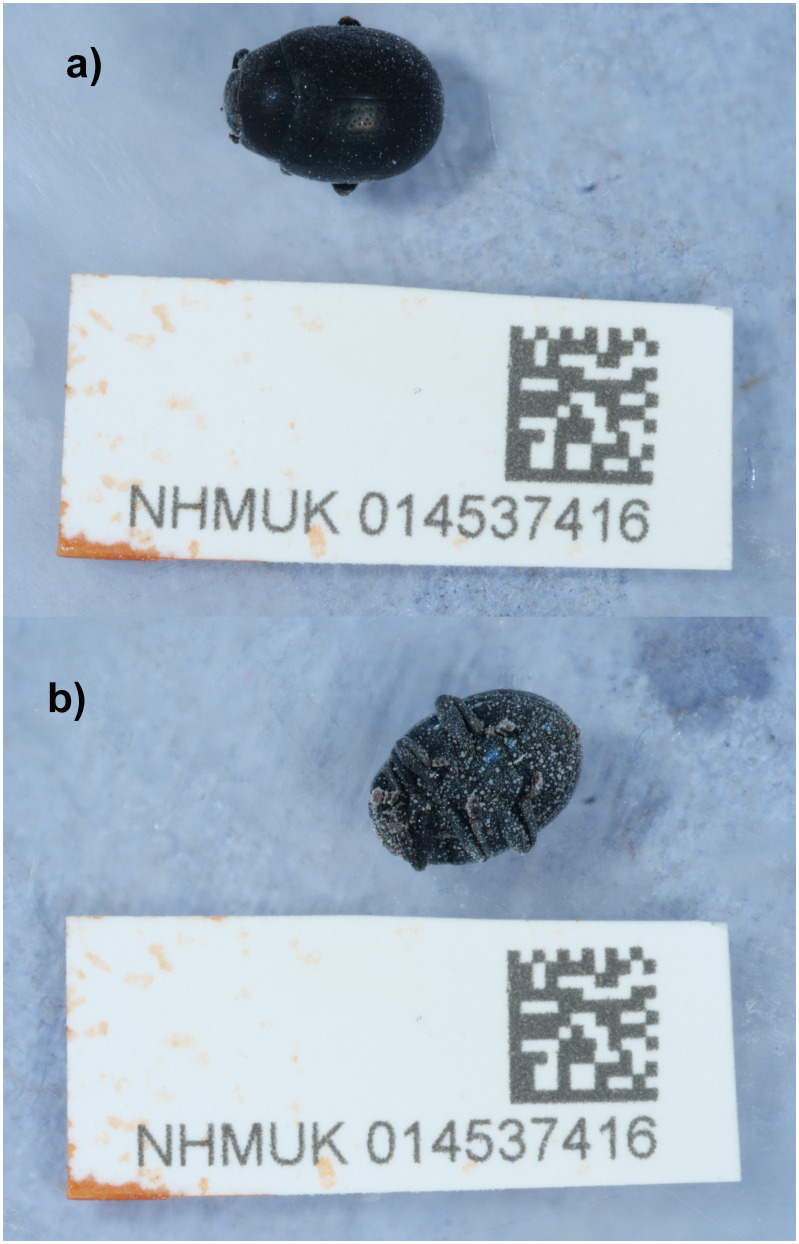
Photograph of the *Chrysolina haemoptera* (icChrHaem1) specimen used for genome sequencing.

The genome of an adult female *Chrysolina haemoptera* ( Figure 1) was sequenced using Pacific Biosciences single-molecule HiFi long reads, generating a total of 20.42 Gb (gigabases) from 2.02 million reads, providing approximately 28-fold coverage. Primary assembly contigs were scaffolded with chromosome conformation Hi-C data, which produced 137.32 Gbp from 909.40 million reads, yielding an approximate coverage of 191-fold. Specimen and sequencing information is summarised in Table 1.

**Table 1.** Specimen and sequencing data for *Chrysolina haemoptera*.

Project information			
**Study title**	*Chrysolina haemoptera*		
**Umbrella BioProject**	PRJEB60718		
**Species**	*Chrysolina haemoptera*		
**BioSample**	SAMEA112221797		
**NCBI taxonomy ID**	75519		
Specimen information			
**Technology**	**ToLID**	**BioSample accession**	**Organism part**
**PacBio long read sequencing**	icChrHaem1	SAMEA112221877	Whole organism
**Hi-C sequencing**	icChrHaem1	SAMEA112221877	Whole organism
Sequencing information			
**Platform**	**Run accession**	**Read count**	**Base count (Gb)**
**Hi-C Illumina NovaSeq 6000**	ERR11040194	9.09e+08	137.32
**PacBio Sequel IIe**	ERR11029703	2.02e+06	20.42

**Figure 2.**
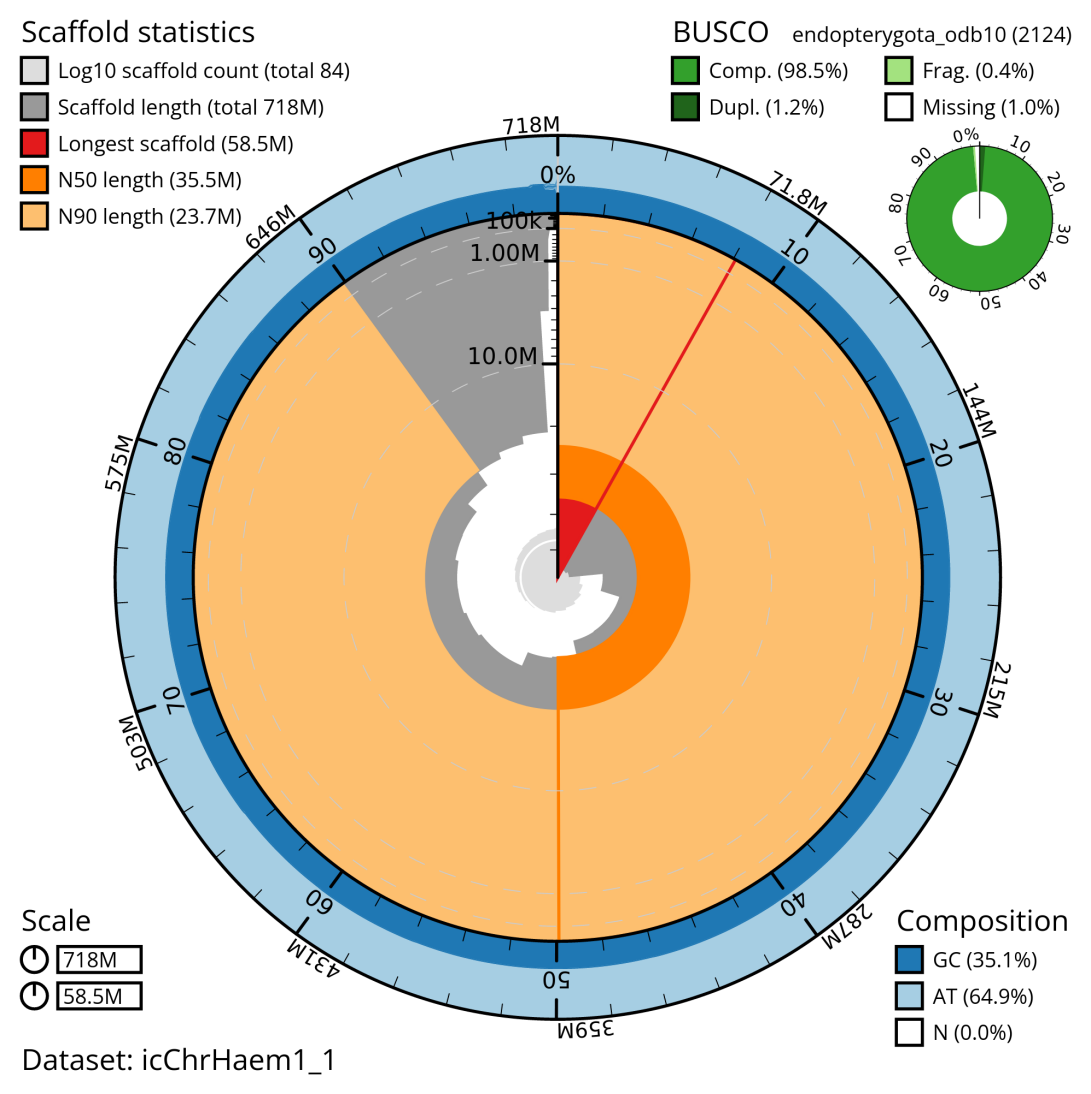
Genome assembly of *Chrysolina haemoptera*, icChrHaem1.1: metrics. The BlobToolKit snail plot shows N50 metrics and BUSCO gene completeness. The main plot is divided into 1,000 size-ordered bins around the circumference with each bin representing 0.1% of the 718,322,849 bp assembly. The distribution of sequence lengths is shown in dark grey with the plot radius scaled to the longest sequence present in the assembly (58,481,741 bp, shown in red). Orange and pale-orange arcs show the N50 and N90 sequence lengths (35,485,444 and 23,672,857 bp), respectively. The pale grey spiral shows the cumulative sequence count on a log scale with white scale lines showing successive orders of magnitude. The blue and pale-blue area around the outside of the plot shows the distribution of GC, AT and N percentages in the same bins as the inner plot. A summary of complete, fragmented, duplicated and missing BUSCO genes in the endopterygota_odb10 set is shown in the top right. An interactive version of this figure is available at https://blobtoolkit.genomehubs.org/view/icChrHaem1_1/dataset/icChrHaem1_1/snail.

**Figure 3.**
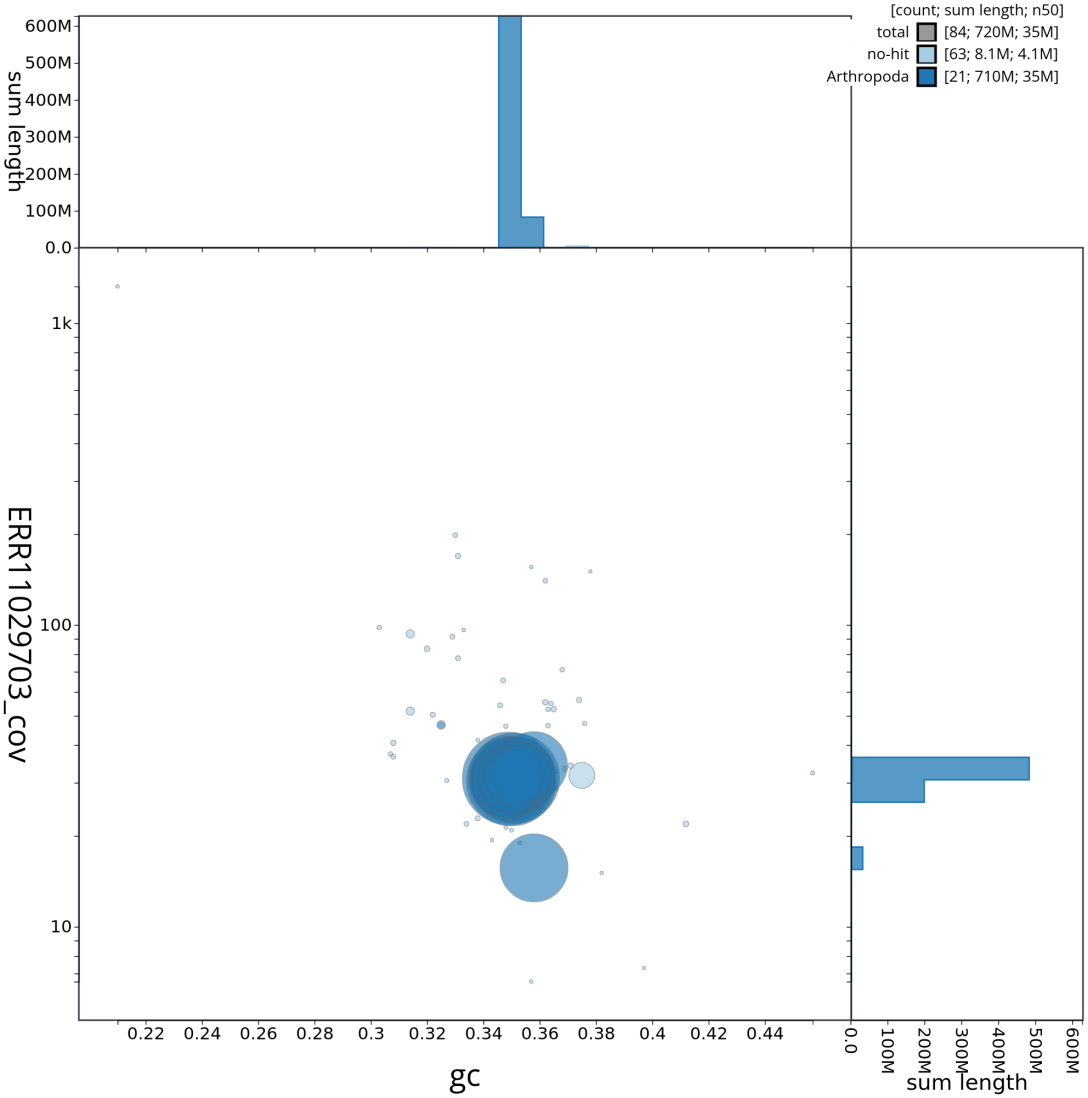
Genome assembly of *Chrysolina haemoptera*, icChrHaem1.1: Blob plot of base coverage in ERR11029703 against GC proportion for sequences in the primary assembly. Sequences are coloured by phylum. Circles are sized in proportion to sequence length. Histograms show the distribution of sequence length sum along each axis. An interactive version of this figure is available at https://blobtoolkit.genomehubs.org/view/icChrHaem1_1/dataset/icChrHaem1_1/blob.

**Figure 4.**
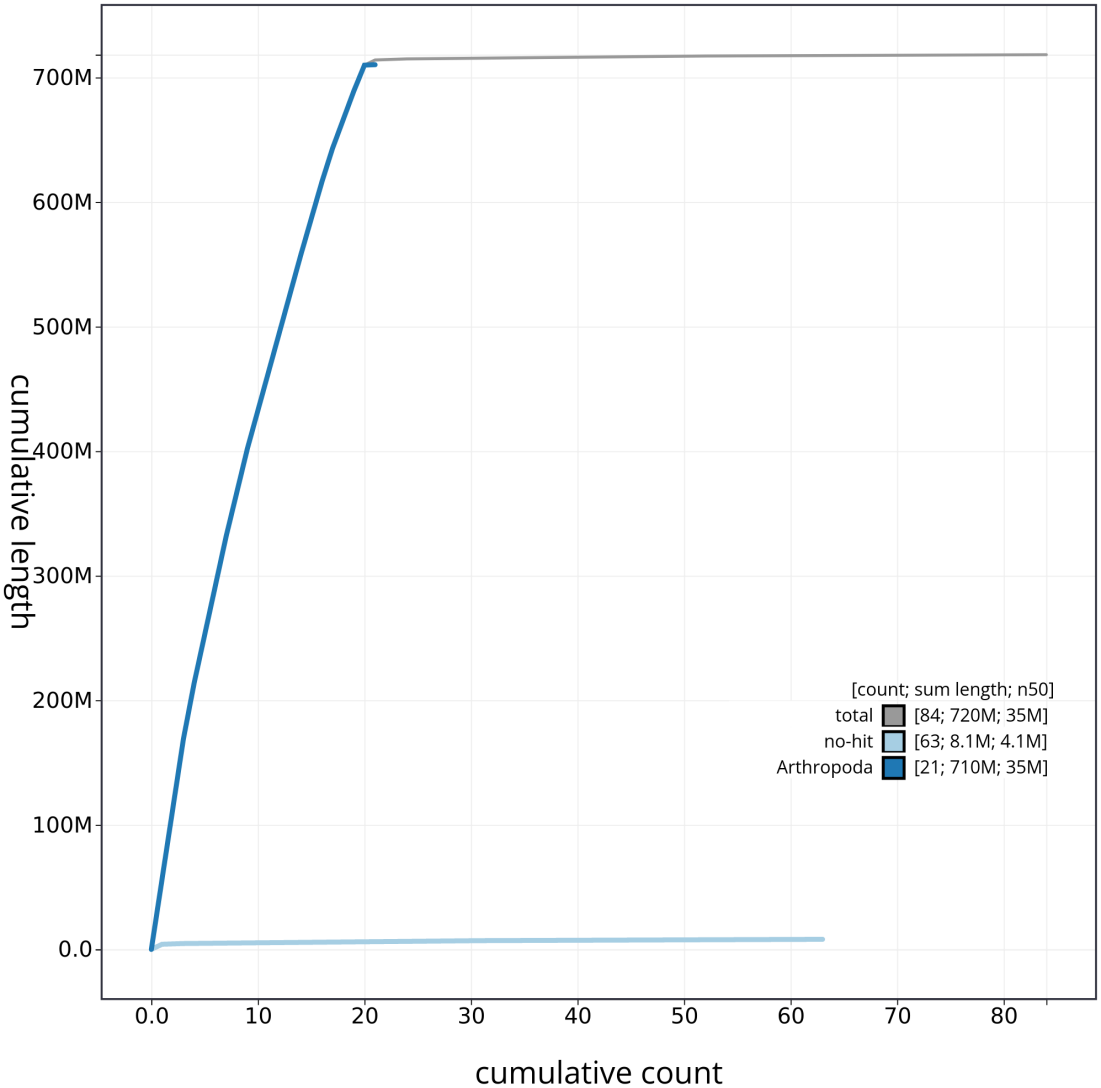
Genome assembly of *Chrysolina haemoptera* icChrHaem1.1: BlobToolKit cumulative sequence plot. The grey line shows cumulative length for all sequences. Coloured lines show cumulative lengths of sequences assigned to each phylum using the buscogenes taxrule. An interactive version of this figure is available at https://blobtoolkit.genomehubs.org/view/icChrHaem1_1/dataset/icChrHaem1_1/cumulative.

**Figure 5.**
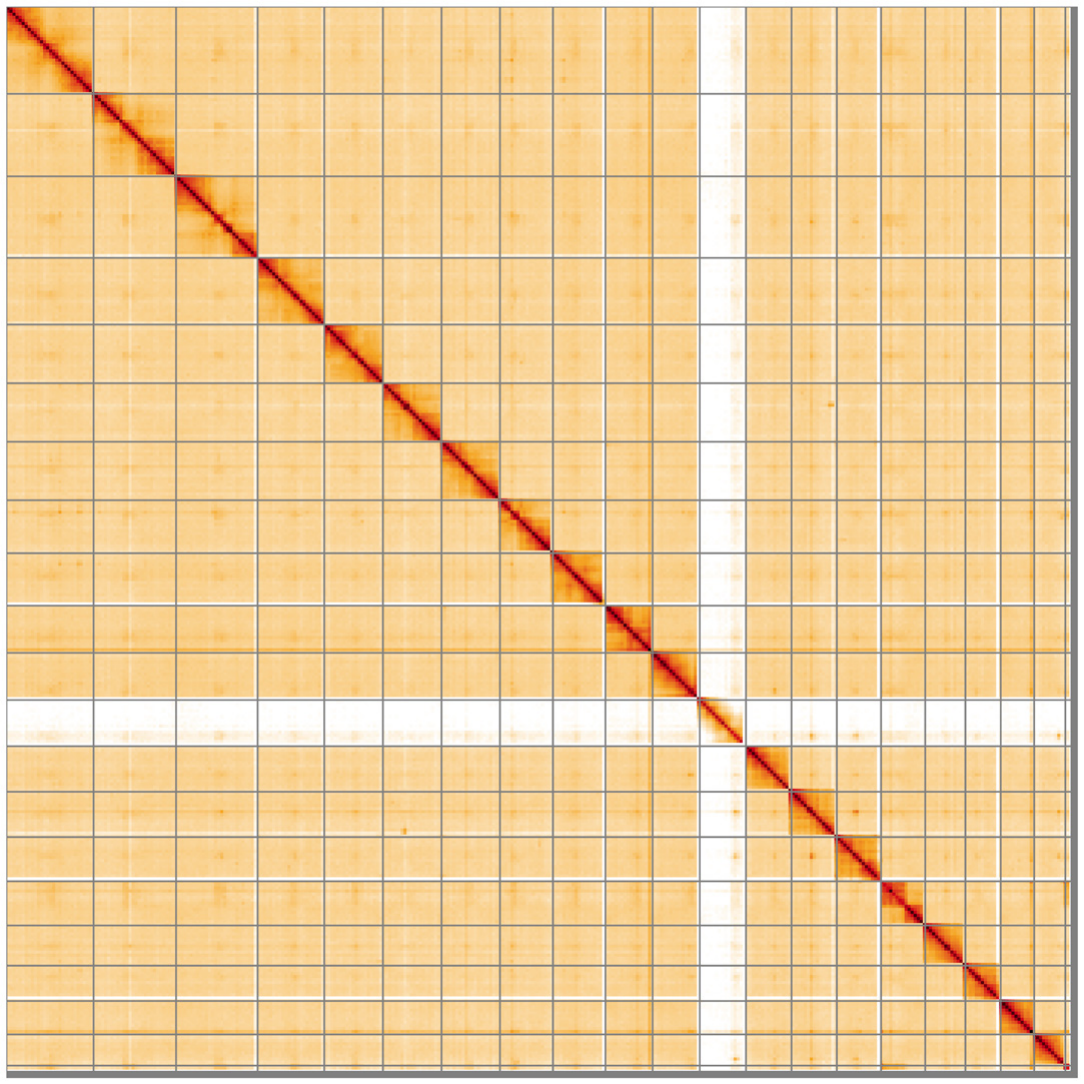
Genome assembly of *Chrysolina haemoptera* icChrHaem1.1: Hi-C contact map of the icChrHaem1.1 assembly, visualised using HiGlass. Chromosomes are shown in order of size from left to right and top to bottom. An interactive version of this figure may be viewed at https://genome-note-higlass.tol.sanger.ac.uk/l/?d=SENHhanRTsiInpiF4InGDw.

Manual assembly curation corrected 48 missing joins or mis-joins and four haplotypic duplications, reducing the assembly length by 0.48% and the scaffold number by 16.83%, and increasing the scaffold N50 by 0.83%. The final assembly has a total length of 718.30 Mb in 83 sequence scaffolds with a scaffold N50 of 35.5 Mb ( Table 2). The total count of gaps in the scaffolds is 462. The snail plot in Figure 2 provides a summary of the assembly statistics, while the distribution of assembly scaffolds on GC proportion and coverage is shown in Figure 3. The cumulative assembly plot in Figure 4 shows curves for subsets of scaffolds assigned to different phyla. Most (99.4%) of the assembly sequence was assigned to 20 chromosomal-level scaffolds, representing 19 autosomes and the X sex chromosome. Chromosome-scale scaffolds confirmed by the Hi-C data are named in order of size ( Figure 5; Table 3). Chromosome X was assigned based on synteny to *Chrysolina oricalcia* (GCA_944452925.2). While not fully phased, the assembly deposited is of one haplotype. Contigs corresponding to the second haplotype have also been deposited. The mitochondrial genome was also assembled and can be found as a contig within the multifasta file of the genome submission.

**Table 2.** Genome assembly data for *Rhimphoctona megacephalus*, iyRhiMega2.1.

Genome assembly		
Assembly name	icChrHaem1.1	
Assembly accession	GCA_958298965.1	
*Accession of alternate haplotype*	*GCA_958298995.1*	
Span (Mb)	718.30	
Number of contigs	546	
Contig N50 length (Mb)	2.2	
Number of scaffolds	83	
Scaffold N50 length (Mb)	35.5	
Longest scaffold (Mb)	58.48	
Assembly metrics*		*Benchmark*
Consensus quality (QV)	59.3	*≥ 50*
*k*-mer completeness	99.99%	*≥ 95%*
BUSCO**	C:98.5%[S:97.3%,D:1.2%], F:0.4%,M:1.0%,n:2,124	*C ≥ 95%*
Percentage of assembly mapped to chromosomes	99.4%	*≥ 95%*
Sex chromosomes	X	*localised homologous pairs*
Organelles	Mitochondrial genome: 17.87 kb	*complete single alleles*
Genome annotation of assembly GCA_958298965.1 at Ensembl		
Number of protein-coding genes	12,298	
Number of non-coding genes	1,376	
Number of gene transcripts	19,677	

* Assembly metric benchmarks are adapted from column VGP-2020 of “Table 1: Proposed standards and metrics for defining genome assembly quality” from Rhie *et al.* (2021).

** BUSCO scores based on the endopterygota_odb10 BUSCO set using version 5.3.2. C = complete [S = single copy, D = duplicated], F = fragmented, M = missing, n = number of orthologues in comparison. A full set of BUSCO scores is available at https://blobtoolkit.genomehubs.org/view/icChrHaem1_1/dataset/icChrHaem1_1/busco.

**Table 3.** Chromosomal pseudomolecules in the genome assembly of *Chrysolina haemoptera*, icChrHaem1.

INSDC accession	Name	Length (Mb)	GC%
OY282584.1	1	58.48	35.0
OY282585.1	2	55.32	35.0
OY282586.1	3	54.66	35.0
OY282587.1	4	44.67	35.0
OY282588.1	5	39.34	35.0
OY282589.1	6	39.29	35.0
OY282590.1	7	39.16	35.0
OY282591.1	8	35.49	35.0
OY282592.1	9	35.19	35.0
OY282593.1	10	29.58	36.0
OY282594.1	11	31.63	35.5
OY282595.1	12	31.63	35.0
OY282597.1	13	30.47	35.0
OY282598.1	14	30.41	35.0
OY282599.1	15	29.66	35.0
OY282600.1	16	26.98	35.0
OY282601.1	17	23.67	35.0
OY282602.1	18	22.4	35.5
OY282603.1	19	20.9	35.0
OY282596.1	X	31.0	36.0
OY282604.1	MT	0.02	21.0

The estimated Quality Value (QV) of the final assembly is 59.3 with *k*-mer completeness of 99.99%, and the assembly has a BUSCO v5.3.2 completeness of 98.5% (single = 97.3%, duplicated = 1.2%), using the endopterygota_odb10 reference set ( *n* = 2,124).

Metadata for specimens, BOLD barcode results, spectra estimates, sequencing runs, contaminants and pre-curation assembly statistics are given at https://links.tol.sanger.ac.uk/species/75519.

## Genome annotation report

The *Chrysolina haemoptera* genome assembly (GCA_958298965.1) was annotated at the European Bioinformatics Institute (EBI) on Ensembl Rapid Release. The resulting annotation includes 19,677 transcribed mRNAs from 12,298 protein-coding and 1,376 non-coding genes ( Table 2; https://rapid.ensembl.org/Chrysolina_haemoptera_GCA_958298965.1/Info/Index). The average transcript length is 15,133.63. There are 1.44 coding transcripts per gene and 5.21 exons per transcript.

## Methods

### Sample acquisition

An adult female *Chrysolina haemoptera* (specimen ID NHMUK014537416, ToLID icChrHaem1) was collected from Loe Bar, Porthleven, England, UK (latitude 50.08, longitude –5.31) on 2021-06-28 using an aerial net. The specimen was collected and identified by Brian Levey (Amgueddfa Cymru) and preserved by dry freezing at – 80°C.

The initial species identification was verified by an additional DNA barcoding process according to the framework developed by Twyford *et al*. (2024). A small sample was dissected from the specimens and stored in ethanol, while the remaining parts of the specimen were shipped on dry ice to the Wellcome Sanger Institute (WSI). The tissue was lysed, the COI marker region was amplified by PCR, and amplicons were sequenced and compared to the BOLD database, confirming the species identification ( Crowley *et al.*, 2023). Following whole genome sequence generation, the relevant DNA barcode region was also used alongside the initial barcoding data for sample tracking at the WSI ( Twyford *et al.*, 2024). The standard operating procedures for Darwin Tree of Life barcoding have been deposited on protocols.io ( Beasley *et al.*, 2023).

### Nucleic acid extraction

The workflow for high molecular weight (HMW) DNA extraction at the WSI Tree of Life Core Laboratory includes a sequence of core procedures: sample preparation and homogenisation, DNA extraction, fragmentation, and purification. Detailed protocols are publicly available on protocols.io ( Denton *et al.*, 2023b).

The sample was prepared in the Tree of Life Core Laboratory: the icChrHaem1 sample was weighed and dissected on dry ice ( Jay *et al.*, 2023). Tissue from whole organism was homogenised using a PowerMasher II tissue disruptor ( Denton *et al.*, 2023a).

HMW DNA was extracted in the WSI Scientific Operations core using the Automated MagAttract v2 protocol ( Oatley *et al.*, 2023). The DNA was sheared into an average fragment size of 12–20 kb in a Megaruptor 3 system with speed setting 31 ( Bates *et al.*, 2023). Sheared DNA was purified by solid-phase reversible immobilisation, using AMPure PB beads to sample to eliminate shorter fragments and concentrate the DNA ( Strickland *et al.*, 2023). The concentration of the sheared and purified DNA was assessed using a Nanodrop spectrophotometer and Qubit Fluorometer using the Qubit dsDNA High Sensitivity Assay kit. Fragment size distribution was evaluated by running the sample on the FemtoPulse system.

### Sequencing

Pacific Biosciences HiFi circular consensus DNA sequencing libraries were constructed according to the manufacturers’ instructions. DNA sequencing was performed by the Scientific Operations core at the WSI on a Pacific Biosciences Sequel IIe instrument. Hi-C data were also generated from remaining tissue of icChrHaem1 using the Arima-HiC v2 kit. The Hi-C sequencing was performed using paired-end sequencing with a read length of 150 bp on the Illumina NovaSeq 6000 instrument.

### Genome assembly, curation and evaluation

***Assembly***

The original assembly of HiFi reads was performed using Hifiasm ( Cheng *et al.*, 2021), with the --primary option. Hi-C reads were mapped to the primary contigs using bwa-mem2 ( Vasimuddin *et al.*, 2019), and the contigs were further scaffolded using the provided Hi-C data ( Rao *et al.*, 2014) in YaHS ( Zhou *et al.*, 2023) using the --break option. Scaffolded assemblies were evaluated using Gfastats ( Formenti *et al.*, 2022), BUSCO ( Manni *et al.*, 2021) and MERQURY.FK ( Rhie *et al.*, 2020).

The mitochondrial genome was assembled using MitoHiFi ( Uliano-Silva *et al.*, 2023), which runs MitoFinder ( Allio *et al.*, 2020) and uses these annotations to select the final mitochondrial contig and to ensure the general quality of the sequence.

***Assembly curation***

The assembly was decontaminated using the Assembly Screen for Cobionts and Contaminants (ASCC) pipeline (article in preparation). Manual curation was primarily conducted using PretextView ( Harry, 2022), with additional insights provided by JBrowse2 ( Diesh *et al.*, 2023) and HiGlass ( Kerpedjiev *et al.*, 2018). Scaffolds were visually inspected and corrected as described by Howe *et al*. (2021). Any identified contamination, missed joins, and mis-joins were corrected, and duplicate sequences were tagged and removed. The sex chromosome was identified by synteny analysis. The entire process is documented at https://gitlab.com/wtsi-grit/rapid-curation (article in preparation).

***Evaluation of the final assembly***

A Hi-C map for the final assembly was produced using bwa-mem2 ( Vasimuddin *et al.*, 2019) in the Cooler file format ( Abdennur & Mirny, 2020). To assess the assembly metrics, the *k*-mer completeness and QV consensus quality values were calculated in Merqury ( Rhie *et al.*, 2020). This work was done using Nextflow ( Di Tommaso *et al.*, 2017) DSL2 pipelines “sanger-tol/readmapping” ( Surana *et al.*, 2023a) and “sanger-tol/genomenote” ( Surana *et al.*, 2023b). The genome was analysed within the BlobToolKit environment ( Challis *et al.*, 2020) and BUSCO scores ( Manni *et al.*, 2021; Simão *et al.*, 2015) were calculated.

The genome evaluation pipelines were developed using the nf-core tooling ( Ewels *et al.*, 2020), use MultiQC ( Ewels *et al.*, 2016), and make extensive use of the Conda package manager, the Bioconda initiative ( Grüning *et al.*, 2018), the Biocontainers infrastructure ( da Veiga Leprevost *et al.*, 2017), and the Docker ( Merkel, 2014) and Singularity ( Kurtzer *et al.*, 2017) containerisation solutions.

Table 4 contains a list of relevant software tool versions and sources.

**Table 4.** Software tools: versions and sources.

Software tool	Version	Source
BlobToolKit	4.2.1	https://github.com/blobtoolkit/blobtoolkit
BUSCO	5.3.2	https://gitlab.com/ezlab/busco
bwa-mem2	2.2.1	https://github.com/bwa-mem2/bwa-mem2
Gfastats	1.3.6	https://github.com/vgl-hub/gfastats
Hifiasm	0.16.1-r375	https://github.com/chhylp123/hifiasm
HiGlass	1.11.6	https://github.com/higlass/higlass
Merqury.FK	d00d98157618f4e8d1a9190026b19b471055b22e	https://github.com/thegenemyers/MERQURY.FK
MitoHiFi	2	https://github.com/marcelauliano/MitoHiFi
PretextView	0.2	https://github.com/wtsi-hpag/PretextView
purge_dups	1.2.3	https://github.com/dfguan/purge_dups
sanger-tol/ascc	-	https://github.com/sanger-tol/ascc
sanger-tol/ genomenote	v1.0	https://github.com/sanger-tol/genomenote
sanger-tol/ readmapping	1.1.0	https://github.com/sanger-tol/readmapping/tree/1.1.0
YaHS	yahs-1.1.91eebc2	https://github.com/c-zhou/yahs

### Genome annotation

The Ensembl Genebuild annotation system ( Aken *et al.*, 2016) was used to generate annotation for the *Chrysolina haemoptera* assembly (GCA_958298965.1) in Ensembl Rapid Release at the EBI. Annotation was created primarily through alignment of transcriptomic data to the genome, with gap filling via protein-to-genome alignments of a select set of proteins from UniProt ( UniProt Consortium, 2019).

### Wellcome Sanger Institute – Legal and Governance

The materials that have contributed to this genome note have been supplied by a Darwin Tree of Life Partner. The submission of materials by a Darwin Tree of Life Partner is subject to the **‘Darwin Tree of Life Project Sampling Code of Practice’**, which can be found in full on the Darwin Tree of Life website here. By agreeing with and signing up to the Sampling Code of Practice, the Darwin Tree of Life Partner agrees they will meet the legal and ethical requirements and standards set out within this document in respect of all samples acquired for, and supplied to, the Darwin Tree of Life Project.

Further, the Wellcome Sanger Institute employs a process whereby due diligence is carried out proportionate to the nature of the materials themselves, and the circumstances under which they have been/are to be collected and provided for use. The purpose of this is to address and mitigate any potential legal and/or ethical implications of receipt and use of the materials as part of the research project, and to ensure that in doing so we align with best practice wherever possible. The overarching areas of consideration are:

- Ethical review of provenance and sourcing of the material
- Legality of collection, transfer and use (national and international)

Each transfer of samples is further undertaken according to a Research Collaboration Agreement or Material Transfer Agreement entered into by the Darwin Tree of Life Partner, Genome Research Limited (operating as the Wellcome Sanger Institute), and in some circumstances other Darwin Tree of Life collaborators.

## Data Availability

European Nucleotide Archive: Chrysolina haemoptera. Accession number PRJEB60718; https://identifiers.org/ena.embl/PRJEB60718 ( Wellcome Sanger Institute, 2023). The genome sequence is released openly for reuse. The *Chrysolina haemoptera* genome sequencing initiative is part of the Darwin Tree of Life (DToL) project. All raw sequence data and the assembly have been deposited in INSDC databases. Raw data and assembly accession identifiers are reported in Table 1 and Table 2.

## Author information

Members of the Natural History Museum Genome Acquisition Lab are listed here: https://doi.org/10.5281/zenodo.12159242.

Members of the Darwin Tree of Life Barcoding collective are listed here: https://doi.org/10.5281/zenodo.12158331

Members of the Wellcome Sanger Institute Tree of Life Management, Samples and Laboratory team are listed here: https://doi.org/10.5281/zenodo.12162482.

Members of Wellcome Sanger Institute Scientific Operations: Sequencing Operations are listed here: https://doi.org/10.5281/zenodo.12165051.

Members of the Wellcome Sanger Institute Tree of Life Core Informatics team are listed here: https://doi.org/10.5281/zenodo.12160324.

Members of the Tree of Life Core Informatics collective are listed here: https://doi.org/10.5281/zenodo.5013541.

Members of the Darwin Tree of Life Consortium are listed here: https://doi.org/10.5281/zenodo.4783558.
