# Supplementary material for: The genome sequence of a leaf beetle, Chrysolina haemoptera (Linnaeus, 1758)
Source: Wellcome Open Res. 2024 Aug 7;9:427. [Version 1] doi: 10.12688/wellcomeopenres.22887.1 (PMC11635191; doi:10.12688/wellcomeopenres.22887.1)
Supplement: Peer review history

# Reviewer report: Reviewer response for version 1

**DOI:** 10.21956/wellcomeopenres.25202.r109941

**Published:** 2024-12-11

**Authors:** Rui-E Nie

**Affiliations:** 1 Anhui Normal University, Wuhu, Anhui Province, China

**Comments:**

 I have carefully read this article. Generally, the article is good. I think it can be indexed after some essential improvements. The quality of the specimen pictures is low. It would be better to provide the images that are clearer and show more morphological traits. In addition, please check some spells such as “most speciose genera” should be “most species genera”.

Are sufficient details of methods and materials provided to allow replication by others?

Partly

Is the rationale for creating the dataset(s) clearly described?

Yes

Are the datasets clearly presented in a useable and accessible format?

Yes

Are the protocols appropriate and is the work technically sound?

Yes

Reviewer Expertise:

The taxonomy，phylogeny and biodiversity of beetles based on the morphological and molecular data.

I confirm that I have read this submission and believe that I have an appropriate level of expertise to confirm that it is of an acceptable scientific standard.

# Reviewer report: Reviewer response for version 1

**DOI:** 10.21956/wellcomeopenres.25202.r101673

**Published:** 2024-10-02

**Authors:** Yu-Hao Huang

**Affiliations:** 1 Guangdong Key Laboratory of Animal Conservation and Resource Utilization, Guangdong Public Laboratory of Wild Animal Conservation and Utilization, Institute of Zoology, Guangdong Academy of Sciences, Guangzhou, China

The data note presents a high-quality, well-annotated chromosome-level genome assembly of the leaf beetle *Chrysolina haemoptera*, which exhibits a BUSCO completeness score of 98.5%. This genome assembly will be valuable for enhancing our understanding of this insect and for research into genetic diversity. The article is clearly written and easy to read, with appropriate sequencing and bioinformatics methods employed. Furthermore, the raw data, assembly, and annotations are accessible.

 I approve of the article in its current form; however, there are several errors and suggestions that could enhance its quality:

 1.There appears to be an error in the species name ( *Rhimphoctona megacephalus*, iyRhiMega2.1) in the title of Table 2. Please correct this and verify whether other species names in the article contain similar errors.

 2.While the link to the annotations is provided in the genome annotation report section, it is advisable to include this link in the data availability section as well, to improve visibility.

 3.It is recommended to provide a BUSCO score for the gene annotations to facilitate user evaluation of their quality.

Are sufficient details of methods and materials provided to allow replication by others?

Yes

Is the rationale for creating the dataset(s) clearly described?

Yes

Are the datasets clearly presented in a useable and accessible format?

Yes

Are the protocols appropriate and is the work technically sound?

Yes

Reviewer Expertise:

comparative genomics, evolutionary biology, bioinformatics, phylogenetics, beetle

I confirm that I have read this submission and believe that I have an appropriate level of expertise to confirm that it is of an acceptable scientific standard.
